# Low Serum Fibroblast Growth Factor 21 Level and Its Altered Regulation by Thyroid Hormones in Patients with Hashimoto’s Thyroiditis on Levothyroxine Substitution

**DOI:** 10.3390/metabo14100565

**Published:** 2024-10-21

**Authors:** Eszter Berta, Sándor Halmi, István Molnár, Dávid Hutkai, Sára Csiha, Harjit Pal Bhattoa, Hajnalka Lőrincz, Sándor Somodi, Mónika Katkó, Mariann Harangi, György Paragh, Endre V. Nagy, Miklós Bodor

**Affiliations:** 1 Division of Metabolism, Department of Internal Medicine, Faculty of Medicine, University of Debrecen, H-4032 Debrecen, Hungary; lorincz_hajnalka@belklinika.com (H.L.); somodi@med.unideb.hu (S.S.); harangi.mariann@med.unideb.hu (M.H.); paragh.gyorgy@med.unideb.hu (G.P.); 2 Department of Clinical Basics, Faculty of Pharmacy, University of Debrecen, H-4032 Debrecen, Hungary; csiha.sara@med.unideb.hu (S.C.); bodor.miklos@med.unideb.hu (M.B.); 3 Doctoral School of Health Sciences, University of Debrecen, H-4032 Debrecen, Hungary; halmi.sandor@med.unideb.hu (S.H.); dr.molnar.istvan@med.unideb.hu (I.M.); 4 Division of Endocrinology, Department of Internal Medicine, Faculty of Medicine, University of Debrecen, H-4032 Debrecen, Hungary; katko.monika@med.unideb.hu (M.K.); endrenagy@med.unideb.hu (E.V.N.); 5 Division of Nephrology, Department of Internal Medicine, Faculty of Medicine, University of Debrecen, H-4032 Debrecen, Hungary; hutkai.david@med.unideb.hu; 6 Kálmán Laki Doctoral School, University of Debrecen, H-4032 Debrecen, Hungary; 7 Department of Laboratory Medicine, Faculty of Medicine, University of Debrecen, H-4032 Debrecen, Hungary; harjit@med.unideb.hu; 8 Department of Emergency Medicine, Faculty of Medicine, University of Debrecen, H-4032 Debrecen, Hungary

**Keywords:** thyroid, fibroblast growth factor 21, FGF21, Hashimoto’s thyroiditis, levothyroxine, hyperlipidemia, hypothyroidism

## Abstract

Background/Objectives: Fibroblast growth factor 21 (FGF21) is a hormonal regulator of lipid and glucose metabolism exerting protection against atherosclerosis by multiple actions on the blood vessels, liver, and adipose tissues. We aimed to investigate serum FGF21 level and its relation to thyroid hormones and metabolic parameters among patients with Hashimoto’s thyroiditis (HT). Methods: Eighty patients with HT on levothyroxine treatment and eighty-two age- and BMI-matched adults without thyroid disease serving as controls were enrolled. Serum FGF21 concentrations were determined with an enzyme-linked immunosorbent assay. Results: Median serum FGF21 level was significantly lower in HT patients compared with controls (74.2 (33.4–148.3) pg/mL vs. 131.9 (44.8–236.3) pg/mL; *p* = 0.03). We found a positive correlation between FGF21 and age, triglyceride, total cholesterol, and low-density lipoprotein cholesterol in both groups, while thyroid stimulating hormone and C-reactive protein showed a positive correlation, and thyroxine had an inverse correlation with FGF21 only in control subjects. According to multiple regression analyses, thyroid status is the main predictor of FGF21 in healthy controls, while it is not a significant predictor of FGF21 among HT patients on levothyroxine supplementation therapy. Conclusions: Our results indicate that the physiological role of thyroid function in the regulation of FGF21 synthesis is impaired in HT patients, which may contribute to the metabolic alterations characteristic of HT patients.

## 1. Introduction

Fibroblast growth factor 21 (FGF21) is a paracrine and endocrine-acting peptide hormone. It plays a role in the regulation of energy homeostasis and body weight. FGF21 belongs to the large family of fibroblast growth factors and exerts functions affecting a broad range of tissues. In mouse models of obesity, FGF21 reduces plasma glucose and triglycerides and promotes weight loss; furthermore, FGF21 overexpression results in resistance to weight gain in mice on high-fat diets [1,2]. Human studies failed to prove the glucose-lowering potential of FGF21, but improvements in dyslipidemia and fatty liver disease have been presented. In humans, FGF21 seems to control macronutrient preference, act as a starvation hormone and as an exercise-induced hepatokine, and serve as a postprandial regulator of metabolism. FGF21 is considered a protective agent from nonalcoholic steatohepatitis and nonalcoholic fatty liver disease [1].

FGF21 is mainly synthesized in the liver, but it is also expressed in the pancreas, skeletal muscle, adipose tissue, hypothalamus, and testis [3,4]. At target tissues, FGF21 binds to and activates members of the FGF-receptor (FGFR) superfamily of receptor tyrosine kinases dependently on the obligate co-receptor βKlotho (KLB), an FGFR-binding, single-pass transmembrane protein [1,4]. FGF21 can be detected in human serum and cerebrospinal fluid [5]. Free fatty acids (FFAs) elevate FGF21 via the activation of the peroxisome proliferator-activated receptor-α (PPAR-α), while FGF21 inhibits lipolysis and FFA production in a fasting state, which suggests an important orchestrating function on the crosstalk between adipose tissue and liver [6]. From a recent study by Nason et al., hepatic FGF-21 seems to be an essential component of glucagon’s weight-loss effects through its central action [7].

Besides its metabolic attributes, FGF21 also seems to have an anti-inflammatory effect, which has been observed in patients with type 2 diabetes mellitus (T2DM) and diabetic peripheral neuropathy [8,9,10], and it seems to ameliorate obesity-related hypothalamic inflammation [4,11]. Circulating FGF21 also modulates immune responses indirectly by influencing the glucose uptake of activated monocytes [12,13].

Circulating levels of FGF21 are increased in subjects with obesity [14], dyslipidemia [15], metabolic syndrome [14], diabetes mellitus [16], nonalcoholic fatty liver disease, coronary artery disease [17], atherosclerosis [18], acute myocardial infarction [19], diabetic nephropathy [20], and arterial hypertension [21]. FGF21 increases with hyperglycemia and predicts the development of diabetes in human studies, and it is also strongly associated with the early stages of nephropathy in T2DM patients. According to these findings, FGF21 might act as an early subclinical indicator of metabolic diseases. The increase in FGF21 in the abovementioned chronic disorders might represent an adaptive process, which, after the extenuation of protective mechanisms and the development of an FGF21-resistant state, fails to induce the desired actions in target tissues [22].

The orchestrating role of FGF21 on metabolism raised the question early of whether a relationship is present between FGF21 and thyroid hormone levels. Hamster experiments found deiodinase 2 (DIO2) upregulated in interscapular brown fat and hypothalamus after FGF21 infusion [23]. DIO2 converts thyroxine (T4) into the biologically active form triiodothyronine (T3). DIO2 is highly expressed in hypothalamic tanycytes, and the consequently altered local concentrations of T3 in the hypothalamus affect appetite and energy expenditure [24]. In animal models, T3 treatment elevated hepatic expression of FGF21 in a PPAR-dependent manner, while the administration of FGF21 reduced serum levels of thyroid hormone, showing a bidirectional relationship [25,26,27]. Although results of animal studies strongly suggest an interaction between FGF21 and thyroid hormones in humans, only limited and unambiguous data are available, as clinical studies found elevated FGF21 levels in both hyperthyroidism and hypothyroidism [28,29,30,31,32,33,34]. Hashimoto’s thyroiditis (HT) is an autoimmune organ-specific disease and the main cause of hypothyroidism, with a global prevalence of 5.8–14.2%. HT is associated with cardiovascular diseases, atherosclerosis, obesity, and thyroid cancer [35,36]. In a recent study by Drongitis et al., the relation between serum FGF21 levels in 30 children and adolescents with subclinical hypothyroidism (SCH) caused by HT compared with serum FGF21 levels of healthy controls was examined. FGF21 levels were not lower in SCH, and their increase was not significant after levothyroxine (LT4) treatment [37].

To date, the relation of serum FGF21 level to metabolic and thyroid status in adult HT patients has not been clarified. Therefore, the aim of our study was to evaluate the relationship between FGF21, thyroid hormone levels, and metabolic parameters among adult patients treated with Hashimoto’s thyroiditis.

## 2. Subjects and Methods

### 2.1. Subjects

We investigated the association between serum FGF21 and lipid and thyroid function parameters in patients followed-up for HT. Eighty Caucasian subjects (HT patients; 75 women and 5 men, mean age: 47 ± 13 years, mean duration of the disease 6.7 ± 4.5 years) and eighty-two Caucasian controls (n = 82, 76 women and 6 men, mean age: 46 ± 14 years) were enrolled at the outpatient clinic of Division of Endocrinology, Department of Internal Medicine, University of Debrecen, Debrecen, Hungary. HT patients received stable levothyroxine substitution treatment for hypothyroidism, and their daily median dose of LT4 was 1.16 µg/kg body weight (interquartile range, IQR: 0.85–1.47). Expectant mothers and patients with concomitant diabetes mellitus, cancer, or concomitant other autoimmune diseases were excluded. The control group consisted of age-, sex-, and BMI-matched controls without documented thyroid disease, whose TSH, fT4, and fT3 levels were within the reference range. This study was approved by the Regional and Institutional Ethics Committee of the University of Debrecen. All patients and controls consented to participate and signed the Informed Consent Form.

### 2.2. Sample Collection and Laboratory Measurements

Venous blood samples were collected into Vacutainer^®^ serum separator tubes and EDTA-anticoagulated tubes (BD-Belliver Industrial Estate, Belliver Way, Roborough, Plymouth PL6 7BP UK) and centrifuged after one-hour coagulation according to local clinical protocol. The plasma and serum were separated by centrifugation at 2200× *g* for 10 min, aliquoted, and stored at −80 °C for later analysis.

Serum FGF21 levels were measured by enzyme-linked immunosorbent assay (ELISA) method (Fibroblast Growth Factor 21 Human ELISA Kit, BioVendor Laboratorni Medicina a.s., Brno, Czech Republic) in accordance with the manufacturer’s instructions.

Serum thyroid hormone levels (free thyroxine-fT4, free triiodothyronine-fT3) and thyroid stimulating hormone (TSH) were measured using electrochemiluminescence immunoassays (FT4 G2 Elecsys, FT3 Elecsys, TSH Elecsys, Roche Diagnostics GmbH, Mannheim, Germany). Reference ranges were 12–22 pmol/L for fT4, 2.4–6.3 pmol/L for fT3 and 0.3–4.2 mU/L for TSH. Antithyroperoxidase (aTPO) antibody concentration was measured by chemiluminescent immunoassay (LIAISON^®^-Anti-TPO, DiaSorin S.p.A., Saluggia, Italy).

Serum high-sensitivity C-reactive protein (hsCRP) was measured by immunoturbidimetric assay; triglyceride, total cholesterol, low-density lipoprotein cholesterol (LDL-C), non-high-density lipoprotein cholesterol (non-HDL-C), and high-density lipoprotein cholesterol (HDL-C) were measured by using enzymatic, colorimetric tests; and glucose was measured by hexokinase kinetic enzymatic assay with a Cobas c600 autoanalyzer (Roche Diagnostics GmbH, Mannheim, Germany).

### 2.3. Statistical Analysis

Statistical analysis was performed by STATISTICA v.14. (Statsoft Inc., Tulsa, OK, USA), and graphs were made using Graphpad Prism v.8. (Graphpad Software, San Diego, CA, USA). Kolmogorov–Smirnov test was used to check the distribution of continuous variables. To compare continuous variables between groups, normally distributed data were analyzed by Student’s unpaired *t*-test, and their data were presented as mean ± standard deviation (SD). Non-normal (or skewed) data were compared using Mann–Whitney U test, and their data were presented with median and 25th and 75th percentiles (interquartile range, IQR). The stochastic relationships of categorical variables were calculated by Chi-square test. Pearson’s correlation was performed for analysis of the relationship between continuous variables. Variables with nonnormal distribution were logarithmically transformed before correlation analysis. Multiple linear regression analyses were performed using log FGF21 as a dependent variable in the HT and control groups separately. Variables correlated with FGF21 in each group in the univariate analyses were included in the multiple regression models. *p*-values below 0.05 were considered statistically significant.

## 3. Results

**Figure 1:**
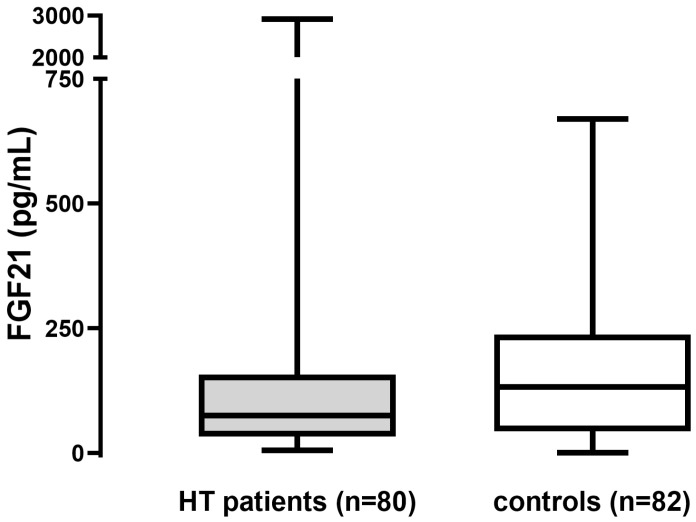
Serum levels of FGF21 in patients with Hashimoto’s thyroiditis (HT) and controls. Box, median, and quartiles; whiskers, minimum, and maximum.

FGF21 levels were significantly lower in the HT patient group than in the age- and BMI-matched controls. TSH and fT4 levels were higher in the HT group than in the controls, while fT3 levels were lower in HT patients than in controls. The serum CRP, total cholesterol, LDL-C, HDL-C, and triglyceride levels were not different between HT patients and the control group, while glucose levels were slightly higher in HT patients compared with controls, but all these parameters were in the normal range (Table 1 and Figure 1).

**Figure 2:**
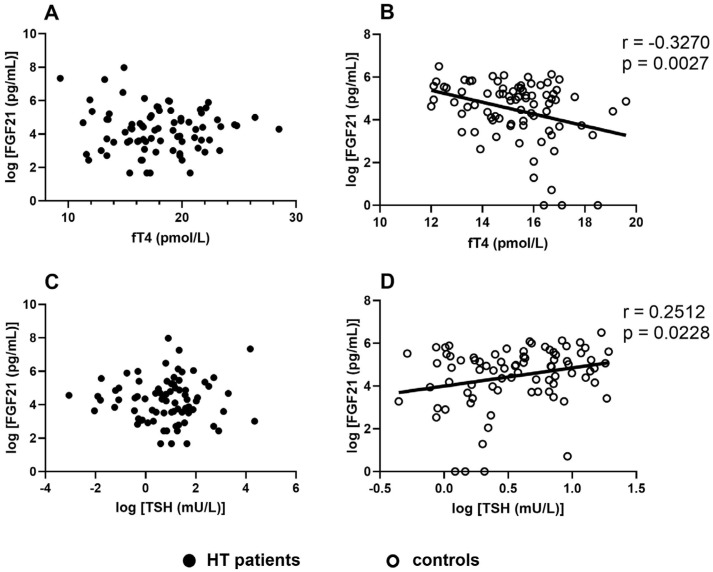
The correlations between the serum free thyroxine (fT4) and FGF21 levels in patients with Hashimoto’s thyroiditis (HT) (**A**) and controls (**B**). The correlations between the serum TSH and FGF21 levels in patients with HT (**C**) and controls (**D**).

FGF21 showed a significant positive correlation with age, triglyceride, total cholesterol, and LDL-C in both studied populations. In HT patients, FGF21 positively correlated with BMI and negatively with HDL-C. In addition, FGF21 was significantly associated with fT4, TSH, and hsCRP, while we failed to find an association between FGF21 and BMI among healthy subjects. Moreover, we did not find significant correlations between thyroid function (fT4 and TSH) and FGF21 in HT patients (Table 2 and Figure 2).

Associations between FGF21 and other studied parameters were further analyzed by multiple linear regression analysis. In HT patients, the model included age, BMI, triglyceride, LDL-C, and HDL-C. According to this analysis, LDL-C concentration was the best predictor of serum FGF21 level (standardized β = 0.225 (0.109); *p* = 0.043) in HT patients. In addition, the other model included age, TSH, fT4, triglyceride, LDL-C, and hsCRP, and fT4 level was the independent predictor of FGF21 among controls (standardized β = −0.270 (0.120); *p* = 0.027) (Table 3).

## 4. Discussion

The relationship between thyroid hormones and FGF21 has not been evaluated thoroughly. In earlier studies, Bonde et al. described normal FGF21 levels in a population of 20 patients with hyperthyroidism, whose free thyroid hormone levels were detected in a wide range [28]. On a larger population of Graves’ disease patients with overt hyperthyroidism, Xiao et al. showed elevated serum FGF21 levels and found a decline after reaching euthyroid hormone levels; simultaneously, the patients’ BMI elevated but remained in the normal range. FGF21 was independently associated with hyperthyroidism [29]. FT3 and FT4 correlated with FGF21 among patients with hyperthyroidism [29,30]. In another study, Bande et al. described the same elevation of FGF21 in hyperthyroidism. The vast majority of the subjects had Graves’ disease, and after treatment, a decline in FGF21 was detected as well [31].

A study conducted by Lee et al. investigated euthyroid individuals and patients with subclinical and overt hypothyroidism and found plasma FGF21 levels to be elevated in patients with hypothyroidism independently of BMI or lipid or glucose metabolism. The cause of hypothyroidism or antibody status was not described [32]. Wang et al. found contradictory data in subclinical and overt hypothyroidism, and a decrease was described in FGF21 levels during the hypofunctional state, with an increase after levothyroxine treatment, and the change in FGF21 levels correlated with an increase in T3 and T4 [33]. Fu et al. investigated healthy subjects and found no relationship between FGF21 and TSH, and FGF21 levels in thyroid autoantibody-positive and -negative groups were not significantly different [34]. The discrepant data on the connection between FGF21 and thyroid function might be partially explained by the hepatic damage resulting in FGF21 elevation being often associated with hyperthyroidism, as clarified in the study of Xiao et al. [30], while in subclinical hypothyroidism, FGF21 might be elevated as an adaptive mechanism to the accompanying obesity [22]. Also, different study designs, population ages, and TSH ranges, together with the technical aspects of different FGF-21 ELISA kits used, might be responsible for the variations in the limited literature sources describing the link between FGF21 and thyroid function.

HT is the most frequent autoimmune disease, with a worldwide prevalence of 7.5%, which is a high ratio, as HT might enhance cardiovascular risk and the development of thyroid cancer [36]. In our study, we aimed to enroll an HT patient group that represents the real-life HT patient population on levothyroxine supplementation. Our patients and the control group were matched for age and BMI, known factors deteriorating metabolism [38,39]. Based on literature data, obesity is associated with HT [35], and overweight and obesity further augment oxidative stress in HT [40,41,42,43,44].

In our study, we detected significantly lower serum FGF21 in HT patients compared with age- and BMI-matched controls. In a previous study, FGF21 levels in children and adolescents with HT-caused subclinical hypothyroidism were similar to controls, although the authors assumed that by increasing the number of patients, the tendency of lower FGF21 in subclinical hypothyroidism and the rise of FGF21 after LT4 treatment could reach statistical significance [37]. Our results seem to make this assumption more feasible and are in line with the work of Wang et al., who detected lower FGF21 among patients with overt hypothyroidism [33].

To the best of our knowledge, we are the first to evaluate the connection between thyroid function and FGF21 in adult HT patients. We found a positive correlation between BMI and FGF21 in the patient population. Furthermore, we found a positive correlation between the TSH and FGF21 levels and an inverse correlation between the fT4 and FGF21 levels in controls, but not in HT patients. The imbalance between the respective thyroid function parameters and FGF21 levels suggests a difference between euthyroid HT on LT4 substitution and healthy subjects regarding the thyroid hormone effects on metabolism, which might contribute to obesity and metabolic changes described among HT patients. After multivariate regression analysis, LDL-C level was the predictor of FGF21 in HT patients, while in controls, FGF21 level was best predicted by fT4, indicating the impaired regulatory role of thyroid hormones in HT. These results may suggest that despite adequate levothyroxine supplementation in HT, the metabolic regulatory imbalance may persist. According to our results, since we could not find a significant correlation between FGF21 and fT3 levels, supplementation of triiodothyronine added to levothyroxine therapy may not improve the FGF21-driven metabolic status of these patients. Furthermore, DIO2 has a short half-life, which is reduced further when its substrate, T4, is present in physiological concentrations. This substrate-driven DIO2 activity serves as a potent regulatory feedback loop when the hypothalamus–hypophysis thyroid axis is intact, but during LT4 supplementation, T3, synthetized by DIO2 in tanycytes and glia cells, might have a blunted effect on metabolism and appetite control [45,46]. Logically, this central regulatory mechanism cannot be repaired by systemic supplementation of T3 due to the short half-life of the presently marketed form. Our results might represent the effect of a potential disruption between thyroid and adipokine regulation of the arcuate nucleus, in which FGF21 might play a role. FGF21 might be linked to other thyroid diseases as well, as higher FGF21 levels are associated with papillary thyroid cancer aggressiveness and might predict the development of Graves’ orbitopathy [47,48]. However, our results, together with the limited data available in the literature, raise the question of whether the length of hypothyroidism, the duration of LT4 supplementation, and the presence of autoimmune processes play a role in FGF21–thyroid interplay.

Our results may explain, at least in part, the clinical observations demonstrating limited efficacy in weight reduction and lipid-lowering, even in adequately treated HT patients. Hence, novel strategies should be considered for metabolic risk reduction tailored to this special patient population. Beneficial FGF21 function has encouraged many attempts to utilize it as a therapeutic agent to treat obesity-related comorbidities, such as type 2 diabetes mellitus and dyslipidemia. Unfortunately, native FGF21 is not appropriate for therapeutic use due to its short half-life and proteolytic cleavage by serum proteases. However, long-acting analogs conjugated to PEG or immunoglobulins, or FGF21 receptor agonists, including bispecific monoclonal antibodies that bind to the FGFR1-KLB complex, as well as avimers (avidity multimers) that activate FGFR1 and KLB, can be considered in the future as targets of drug development [49]. Glucagon-like peptide-1 receptor agonists (GLP-1RAs) are emerging as first-line pharmacological treatments for sustained weight loss, also exerting lipid-lowering effects [50]. However, these agents exert their circulating FGF21 lowering effect only when combined with exercise [51]. Therefore, the complex metabolic effects of GLP-1RA therapy in HT patients should also be studied to clarify the exact effect of GLP-1RA therapy in this patient population. It must be noted that other antidiabetic, lipid-lowering, and blood-pressure-lowering agents did not affect the serum FGF21 levels in diabetes mellitus [52].

Some limitations of the study must be mentioned, including the relatively low number of subjects. The evaluation of the effect of oxidative stress on FGF21 levels during the development of hypothyroidism and after starting levothyroxine treatment in HT seems to be a promising scientific target. Also, the investigation of FGF21 levels in HT patients with known obesity, metabolic syndrome, cardiovascular disease, and obesity is necessary to clarify the effect of lower FGF21 on the incidence of the mentioned pathologies. Moreover, prospective studies of HT patients without concomitant cardiovascular or metabolic disorders could reveal the potential role of FGF21 in the development of cardiovascular events or metabolic abnormalities. However, our patient group had been followed and treated for years, which might add to the strength of the results.

## 5. Conclusions

The relationship between thyroid hormones and FGF21 is not yet understood in detail, and the role of FGF21 in the development of thyroid diseases and consequent metabolic dysfunction and weight gain needs to be further elucidated. In our study, we found lower serum FGF21 levels, with a lack of correlation with thyroid function in HT patients, which might be related to a change in metabolic risk. Our results and assumptions need to be tested on a larger patient population.

## Figures and Tables

**Table 1:** Anthropometric and laboratory parameters of HT patients and controls. Values are presented as mean ± standard deviation or median (lower quartile, upper quartile).

	HT Patients (n = 80)	Controls (n = 82)	*p*
Age (years)	47 ± 13	46 ± 14	0.610
BMI (kg/m^2^)	25.5 (23.9–30.5)	26.1 (22.2–30.0)	0.997
FGF21 (pg/mL)	74.2 (33.4–148.3)	131.9 (44.8–236.3)	0.030
hsCRP (mg/L)	1.7 (1.0–4.5)	2.6 (0.9–5.5)	0.268
TSH (mIU/L)	2.80 (1.03–4.79)	1.77 (1.25–2.38)	0.004
fT4 (pmol/L)	17.9 ± 3.7	15.3 ± 1.7	<0.0001
fT3 (pmol/L)	4.56 ± 0.62	5.04 ± 0.62	<0.0001
Glucose (mmol/L)	5.2 (4.9–5.6)	5.1 (4.8–5.6)	0.039
Triglyceride (mmol/L)	1.4 (0.9–2.0)	1.2 (0.9–1.7)	0.058
Total cholesterol (mmol/L)	5.3 ± 1.1	5.4 ± 1.1	0.629
LDL-C (mmol/L)	3.2 (2.6–4.1)	3.3 (2.7–4.1)	0.672
HDL-C (mmol/L)	1.5 (1.3–1.8)	1.5 (1.2–1.7)	0.539

Abbreviations: BMI: body mass index, FGF21: fibroblast growth factor 21, fT4: thyroxine, fT3: triiodothyronine, HDL-C: high-density lipoprotein cholesterol, hsCRP: high-sensitivity C-reactive protein, HT: Hashimoto’s thyroiditis, LDL-C: low-density lipoprotein cholesterol, TSH: thyroid stimulating hormone.

**Table 2:** Correlations between FGF21 and age, BMI, thyroid hormones, lipid parameters, and hsCRP in Hashimoto’s thyroiditis patients and healthy controls.

	HT Patients (n = 80)		Controls (n = 82)	
Parameters	r	*p*	r	*p*
Age (years)	0.403	<0.001	0.317	0.004
BMI (kg/m^2^)	0.373	0.001	0.200	0.071
TSH	0.005	0.967	0.251	0.023
fT4 (pmol/L)	−0.071	0.530	−0.327	0.003
fT3 (pmol/L)	−0.163	0.148	−0.022	0.843
Glucose (mmol/L)	0.138	0.290	0.188	0.091
Triglyceride (mmol/L)	0.395	<0.001	0.344	0.003
Total cholesterol (mmol/L)	0.350	0.002	0.291	0.012
LDL-C (mmol/L)	0.358	0.001	0.323	0.006
HDL-C (mmol/L)	−0.242	0.033	−0.186	0.117
hsCRP (mg/L)	0.211	0.073	0.260	0.019

Abbreviations: BMI: body mass index, FGF21: fibroblast growth factor 21, fT4: thyroxine, fT3: triiodothyronine, hsCRP: high-sensitivity C-reactive protein, HT: Hashimoto’s thyroiditis, LDL-C: low-density lipoprotein cholesterol, non-HDL-C: non-high-density lipoprotein cholesterol, TSH: thyroid stimulating hormone.

**Table 3:** Predictors of FGF21 level in HT patients and controls.

	HT Patients		Controls	
Predictors	st. β (SE of st. β)	*p*	st. β (SE of st. β)	*p*
Age	0.223 (0.118)	0.063	0.094 (0.131)	0.476
BMI	0.232 (0.121)	0.059	na	na
TSH	na	na	0.165 (0.114)	0.155
fT4	na	na	−0.270 (0.120)	0.027
Triglyceride	0.119 (0.131)	0.365	0.147 (0.144)	0.310
LDL-C	0.225 (0.109)	0.043	0.152 (0.157)	0.337
HDL-C	−0.096 (0.109)	0.465	na	na
hsCRP	na	na	0.075 (0.118)	0.337

Abbreviations: BMI: body mass index, FGF21: fibroblast growth factor 21, fT4: thyroxine, hsCRP: high-sensitivity C-reactive protein, HT: Hashimoto’s thyroiditis, HDL-C: high-density lipoprotein cholesterol, LDL-C: low-density lipoprotein cholesterol, TSH: thyroid stimulating hormone, na: not applicable.

## Data Availability

Data is contained within the article.
